# Evaluation of Infectivity, Virulence and Transmission of FDMV Field Strains of Serotypes O and A Isolated In 2010 from Outbreaks in the Republic of Korea

**DOI:** 10.1371/journal.pone.0146445

**Published:** 2016-01-06

**Authors:** Juan M. Pacheco, Kwang-Nyeong Lee, Michael Eschbaumer, Elizabeth A. Bishop, Ethan J. Hartwig, Steven J. Pauszek, George R. Smoliga, Su-Mi Kim, Jong-Hyeon Park, Young-Joon Ko, Hyang-Sim Lee, Dongseob Tark, In-Soo Cho, Byounghan Kim, Luis L. Rodriguez, Jonathan Arzt

**Affiliations:** 1 Plum Island Animal Disease Center, Foreign Animal Disease Research Unit, Agricultural Research Service, United States Department of Agriculture, Plum Island, New York, United States of America; 2 Animal and Plant Quarantine Agency, Department of Animal and Plant Health Research, Anyang-si, Gyeonggi-do, Republic of Korea; 3 Oak Ridge Institute for Science and Education, PIADC Research Participation Program, Oak Ridge, Tennessee, United States of America; The Pirbright Institute, UNITED KINGDOM

## Abstract

Since the early 2000s outbreaks of foot-and-mouth disease (FMD) have been described in several previously FMD-free Asian nations, including the Republic of Korea (South Korea). One outbreak with FMD virus (FDMV) serotype A and two with serotype O occurred in South Korea in 2010/2011. The causative viruses belonged to lineages that had been spreading in South East Asia, far East and East Asia since 2009 and presented a great threat to the countries in that region. Most FMDV strains infect ruminants and pigs, as it happened during the outbreaks of FMDV serotype O in South Korea. Contrastingly, the strain of serotype A affected only ruminants. Based upon these findings, the intention of the work described in the current report was to characterize and compare the infectivity, virulence and transmission of both strains under laboratory conditions in cattle and pigs, by direct inoculation and contact exposure. As expected, FMDV serotype O was highly virulent in both cattle and swine by contact exposure and direct inoculation. Surprisingly, FMDV serotype A was highly virulent in swine, but was less infectious in cattle by contact exposure to infected swine or cattle. Interestingly, similar quantities of aerosolized FMDV RNA were detected during experiments with viruses of serotypes O and A. Specific virus-host interaction of A/SKR/2010 could affect the transmission of this strain to cattle, and this may explain in part the limited spread of the serotype A epizootic.

## Introduction

Foot and mouth disease (FMD) is a highly contagious and economically devastating viral disease of cloven hoofed animals. Three serotypes of FMD virus (FMDV), O, A and Asia1 are endemic in much of Asia including Central and South East Asia. FMDV is one of the most dreaded ailments of livestock due to its broad host range [1, 2] and high rate of contagious spread by direct or indirect contact, including airborne transmission. In contrast, some isolates exhibit a more restricted host range including the virus isolated in Taiwan in 1997 [3].

Efforts to control FMD outbreaks in previously free regions result in substantial impact upon regional and international trade practices and economic stability. Several recent historical examples of this effect include outbreaks in FMD-free countries including Argentina, Brazil, Uruguay, Japan, Republic of Korea (hereafter referred to as South Korea), Bulgaria, and Western Europe in the 2000s [4–9].

Subsequent to an outbreak in 1934, South Korea remained FMD-free for 66 years. Since 2000 six FMD epidemics have been recorded (in March 2000, May 2002, January 2010, April 2010, November 2010–April 2011 [10] and during 2014, in July (OIE, 2014. Foot and mouth disease, Republic of Korea. Immediate notification, 24/07/2014) and December (OIE, 2014. Foot and mouth disease, Republic of Korea. Immediate notification, 05/12/2014). In January-March 2010 an epidemic of FMDV serotype A occurred in the Northwest region of the country. This virus belonged to the Asia topotype that had been prevalent in Southeast Asia since 2008. This was a limited outbreak, that involved only 7 farms in a radius less of 10 km, and affected only cattle (clinically) and deer (subclinically), but not pigs. Control was rapidly achieved by means of culling and movement restrictions and the country was declared FMD-free on March 23, 2010 [11]. Two weeks after declaration of FMD eradication an epidemic of FMDV serotype O was reported. This one lasted from April to June of 2010, with 13 outbreaks, including cases in pigs, cattle, and goats (clinically) and wild boar (subclinically). The new incursion strain belonged to the South-East Asian topotype of serotype O. Control was achieved by stamping out and on September 27, 2010 the country regained FMD-free status [10]. Less than two months later, another serotype O outbreak occurred, that lasted from November 2010 to April 2011. The virus belonged to the same topotype described for the previous incursion, but this outbreak, the biggest ever in South Korea, involved 3748 farms of cattle, pigs, goats and deer distributed throughout most of the country. The epidemic was controlled by stamping out and vaccination [12].

Considering the apparent differences in host range observed in the field across the 2010/2011 Korean outbreaks, the main objective of the current work was to investigate and compare the infectivity, virulence, air shedding, and transmission of serotype A and O field strains which caused the outbreaks. This was achieved by conducting a series of time course studies in which steers or pigs were directly inoculated with either strain and naïve animals were brought into contact for a limited exposure time. Results were ultimately interpreted in the context of data collected in the field during the outbreaks. For serotype O, we confirmed the similar susceptibility of both species to this strain. In contrast to field data, in which serotype A affected only cattle, our study found this strain to cause more severe disease in pigs than in cattle.

## Materials and Methods

### Virus strains and cell cultures

FMDV A/Pocheon/01/KOR/2010, NVRSQ01, isolate 201001_01V, corresponds to vesicular fluid collected on January 2010 from a bovine with clinical FMD from Pocheon, Gyeonggi province [11], and it will be referred to as A/SKR/2010 hereafter. FMDV O/PJ/KOR/2010, NVRQS10, isolate 201012_49V, corresponds to vesicular fluid collected on December 2010 from a bovine with clinical FMD from Paju County, Gyeonggi province [12], and it will be referred as O/SKR/2010 hereafter. The cell line used for virus titrations was LFBK-αvβ6 [13].

### FMDV stock production

Viruses were amplified only in the species they were isolated from, bovine for serotype A and bovine and porcine for serotype O. In order to produce a bovine-amplified stock of each inoculum for both viral strains, serotype A and O, two steers per strain were inoculated with 10^5.30^ plaque forming units (PFU) of one field-collected vesicular fluid by the intraepithelial lingual route [14]. Sloughed epithelium from the tongue was harvested 24 h post inoculation (hpi), pooled, macerated, diluted 1:50 in MEM with 25 mM Hepes, clarified, filtered (0.45 μm filter), aliquoted in 1-ml tubes and frozen at -70°C until further titration and usage. Porcine-amplified stock of serotype O inoculum was generated as previously described [15]. Briefly, four pigs were inoculated by the intradermal route in the heel bulb [16] with 10^5.00^ PFUs each. When vesicles were visualized, at 48–72 hpi, pigs were deeply sedated to harvest vesicular fluid from FMD lesions at coronary bands and interdigital spaces. Vesicular fluid was collected in tubes and an equal volume of MEM with 25 mM HEPES was added to each tube before freezing at -70°C. After harvesting all, vesicular fluids were thawed, pooled, diluted 1:50 in MEM with 25 mM HEPES, clarified, filtered (0.45 μm filter), aliquoted and stored at -70°C. One aliquot of each viral stock was subsequently thawed for titration in cells and to inoculate animals

### Animal experiments

#### Ethics and animal care

All animal procedures were performed following Protocol 231-R-11, approved by the Plum Island Animal Disease Center Institutional Animal Care and Use Committee (IACUC), which ensured ethical and humane treatment of experimental animals. This included daily monitoring of the health of the experimental animals. Whenever excessive pain (determined by moderate or more severe lameness, vesicles detected on the feet/tongue, or decreased appetite) or temperature >40.3°C was observed, steps were taken to minimize animal suffering by delivery of analgesics and/or anti-inflammatory drugs at 1.1–2.2 mg per kg of flunixin meglumine every 12–24 h and/or 0.1 mg/kg of butorphanol tartrate every 8–24 h. If pain could not be pharmacologically controlled, animals were humanely euthanized. For virus direct-inoculation, steers were sedated using xylazine, IM, 0.22 mg/kg. Sedation was reversed with tolazoline, IV, 2 mg/kg. For virus direct-inoculation in pigs, animals were sedated with a combination of 3 mg/kg telazol, 8 mg/kg ketamine, and 4 mg/kg xylazine. Euthanasia for both species was performed utilizing Fatal-plus, IV, 10 ml/45.3 kg after sedated as indicated above. Twenty four Holstein steers, 9–12 months old, weighing 200–250 kg, and twenty eight castrated male Yorkshire pigs, weighing 25–30 kg were obtained from an AAALAC-accredited experimental-livestock provider (Thomas-Morris Inc., Reisterstown, MD). For all experiments, animals were housed in groups as described below in a BSL-3-Ag animal facility from time of inoculation until time of euthanasia.

#### Direct inoculation of steers and steer-to-steer, steer-to-pig contact challenge

**Fig 1:**
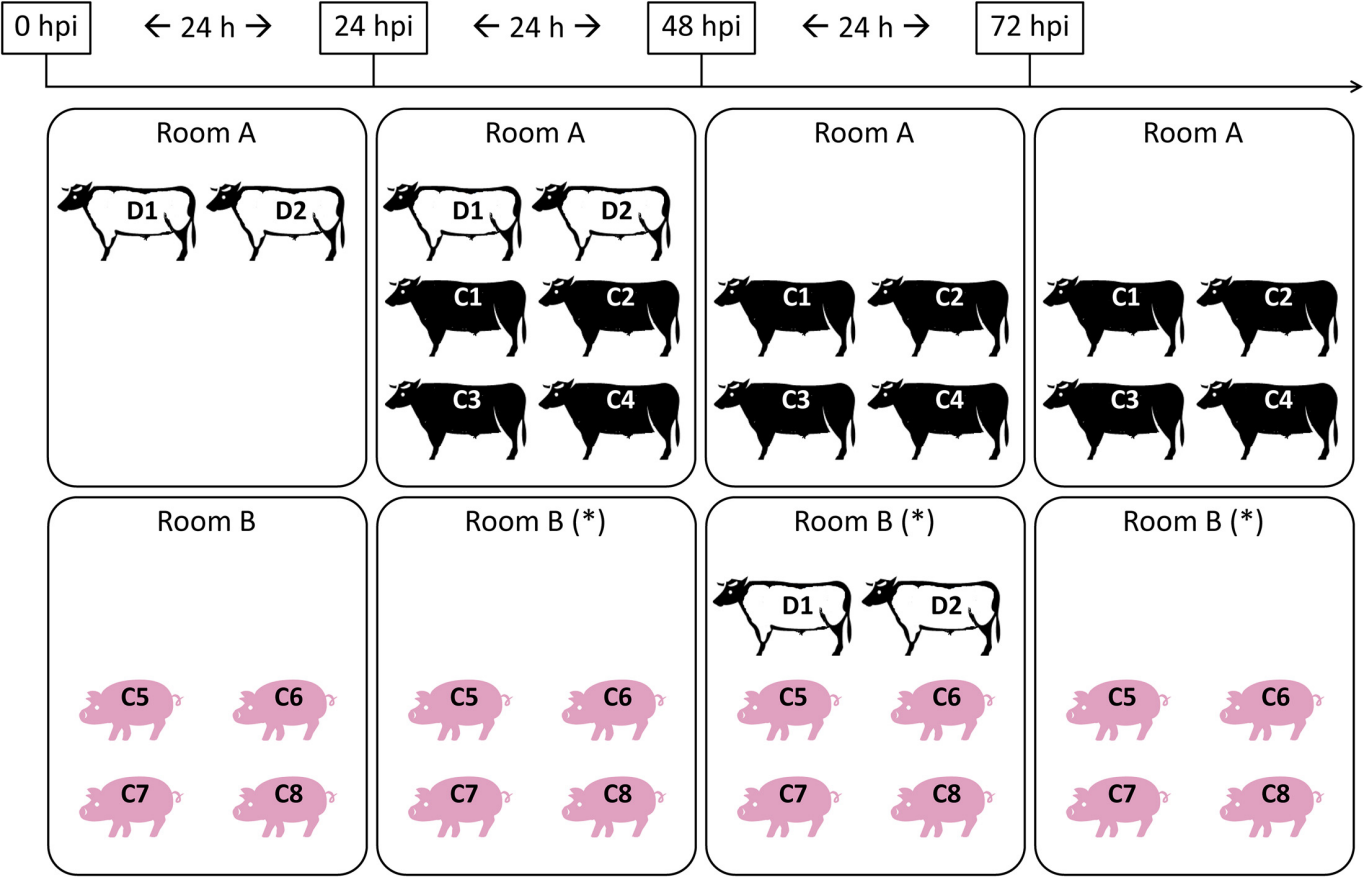
Experimental design for two experiments performed with FMDV A/SKR/2010 and O/SKR/2010. White steers (D1-D2) represent direct-inoculated animals, used as donors for contact exposure later. Black steers (C1 to C4) and pigs (C5 to C8) represent contact-exposed animals. Timeline on top, relative to inoculation of donors, is expressed in hours post inoculation (hpi). Contact started at 24 h post inoculation and lasted for 24 h for each period. (*) Room from where air samples were collected

The contact experiments were designed in order to conduct two immediately consecutive 24-h contact exposures using the same donors but different recipients. All contact exposures were performed maintaining a 1:2 donor to recipient ratio (Fig 1). For each strain, direct inoculation was performed in two steers by intraepithelial lingual administration (10^6.00^ PFU for serotype O and 10^6.80^ PFU for serotype A) of bovine-amplified stock. At 24 hpi, these 2 direct-inoculated (DI) steers and 4 naïve steers were brought together into the same room (room A, Fig 1), sharing an area of 12 m^2^ to achieve 24 h of direct contact. Animals were fed daily and watered *ad libitum*. After the 24 h period (at 48 hpi), steer-to-steer direct contact ended, and the two DI steers were brought to share a room with 4 naïve pigs (room B, Fig 1), to perform steer-to-pig contact for 24 h, in a space of 12 m^2^. During this period, both species were fed with cattle feed (alfalfa cubes) spread on the floor, to increase animal interaction, and water was administered *ad libitum*. At the end of this contact period of 24 h (72 hpi), DI steers were removed from the room. All 4 contact-exposed (CE) steers were kept together in direct contact with one another in an area of 12 m^2^ and monitored for 7 days after contact started. Similarly, all 4 CE pigs were kept together in direct contact in an area of 12 m^2^ and monitored for 7 days after contact started. During daily sample collection and clinical evaluation, the same group of researchers entered both rooms, always going from the room housing steers to the room housing pigs.

#### Direct inoculation of pigs and pig-to-pig, pig-to-steer contact challenge

**Fig 2:**
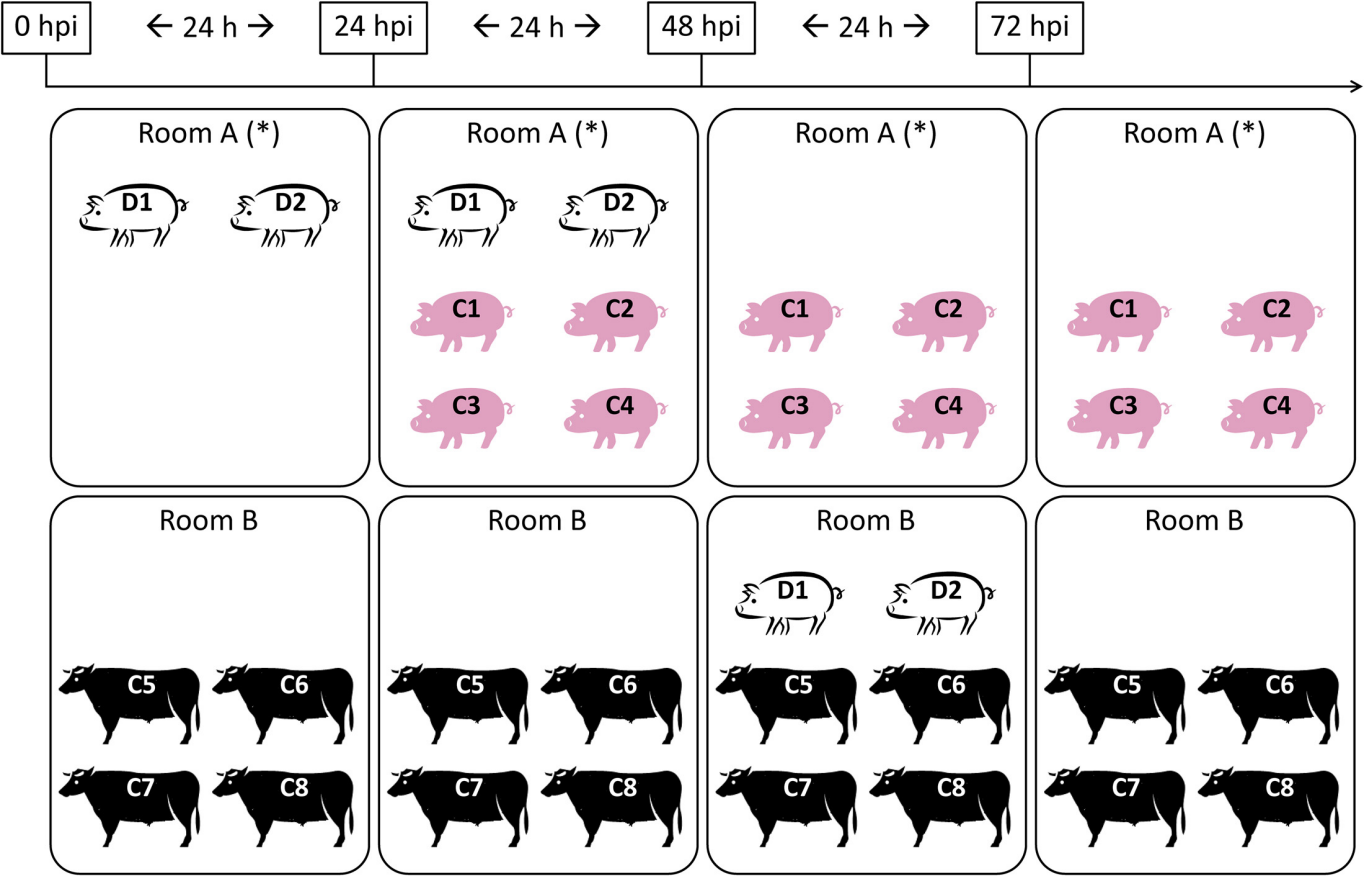
Experimental design of two experiments performed with FMDV A/SKR/2010 and O/SKR/2010. White pigs (D1-D2) represent direct-inoculated animals, used as donors for contact exposure later. Pink pigs (C1 to C4) and steers (C5 to C8) represent contact-exposed animals. Timeline on top, relative to inoculation of donors, is expressed in hours post inoculation (hpi). Contact started at 24 h post inoculation and lasted for 24 h for each period. (*) Room from where air samples were collected

Two experiments using pigs as donors were performed similarly as described above for experiments in which steers were used as donors with few modifications (Fig 2). In these experiments, two donor pigs were inoculated intradermally in the heel bulb with 10^5.00^ PFU of porcine-amplified stock for serotype O or 10^7.80^ PFU of bovine-amplified stock for serotype A. Contact exposure occurred first pig-to-pig (from 24 to 48 hpi, Room A in Fig 2) and then with pig-to-steer (from 48 to 72 hpi, Room B in Fig 2). During pig-to-pig contact, feed was withheld to increase interaction, but water was available *ad libitum*. During pig-to-steer contact, animals were fed with alfalfa cubes as described above. CE pigs were followed for 7 days and CE steers were followed for 7 to 15 days. During daily sample collection and clinical evaluation, the same group of researchers entered both rooms, always going from the room housing pigs to the room housing steers.

#### Clinical evaluation and sampling

Clinical observations and sample collection were performed daily from 0 to 72 hpi for DI animals and from 0 to 168 or 360 h post exposure (hpe) for CE animals. Blood and nasal swabs were collected from both species, whereas tonsil swabs were collected only from pigs. Swabs were collected with cotton-tipped swabs and then immersed in 2 ml of MEM with 25 mM HEPES. Serum was separated from blood collected from the jugular vein. All samples were frozen at -70°C immediately after collection. Clinical signs in pigs were scored as previously described [17] with a maximum potential score of 20 points for CE pigs and 16 for DI pigs since the inoculated foot was not used for scoring. Clinical signs in steers were determined using a maximum possible score of 5 points for CE steers (1 point per affected foot plus one point for vesicles anywhere in the head) and 4 for DI steers, since the head vesicles were not used for scoring.

#### Air samples

Air samples were collected daily from rooms that held CE pigs, starting at 24 hpi in room B for experiments that utilized steers as donors (see asterisks in Fig 1) and at 0 hpi in room A for experiments that utilized pigs as donors (see asterisks in Fig 2). Fig 1 shows that only 4 naïve pigs were in room B where air was being collected until 48 hpi when two DI steers were moved into the room. At 72 hpi, steers were removed from the room and only the 4 CE pigs remained in the room until the end of sample collection. Fig 2 shows that 2 DI pigs were in room A from 0 to 48 hpi, while 4 naïve pigs were moved into the room starting at 24 hpi and remained in the room until the end of the experiment. Air sampling was performed as previously described [15] by using a Dry Filter Unit (DFU) Model 1000 air pump developed by the Program Executive Office for Chemical Biological Defense (PEO-CBD), holding two separate Lockheed Martin polyester filter disks (1.0 um filter, diameter 47 mm, Catalogue number DFU-P-24, Lockheed Martin). The air flow for this system was 8673 L/h. Filters were replaced every 24 h and immediately stored at -70°C until further processing.

### FMDV RNA detection

Samples were thawed and processed for RNA extraction and rRT-PCR as previously described for serum and swabs [18, 19] or air filters [15]. FMDV RNA copy numbers per milliliter of fluid (serum and swabs) or per 1000 l of air (air samples) were calculated based on a O/SKR/2010- or A/SKR/2010-specific calibration curve developed with *in vitro*-synthesized FMDV RNA obtained from FMDV-3D plasmids generated from the original isolates. For this purpose, 10-fold serial dilutions of a known amount of FMDV RNA were made in nuclease-free water and each dilution was tested in triplicate by FMDV rRT-PCR. For swabs, collected in media, dilution factors were used to correct to the original FMDV RNA concentration of the secretion.

### Statistical analysis

The area under the curve (AUC) was calculated for the clinical scores of each animal as well as for the log_10_-scaled FMDV RNA concentrations in serum and nasal swabs using the trapezoidal method. Before the AUC calculation, clinical scores were normalized by dividing by the maximum possible score, and RNA concentrations were adjusted by subtracting the limit of detection (LOD) value from all observations. Individual AUCs between groups were compared by non-parametric Mann-Whitney U tests. P-values <0.05 were considered significant. For each animal, the first day with a (non-normalized) clinical score >1 was considered the onset of clinical disease. In each group, a mean time to onset (in days) and a 95% confidence interval for that mean were calculated. The association between virus shedding and its detection in room air was tested by calculating Pearson’s product-moment correlation coefficients for FMDV RNA concentrations in nasal or tonsil swabs and in filter samples.

## Results

### Master stock preparation

Serotype O strain was amplified in steers and pigs, bovine- and porcine-amplified stocks were named O/SKR/2010-PI-BovP1 and O/SKR/2010-PI-PorP1, with titers (performed in LFBK-αvβ6) of 10^9.50^ PFU/ml and 10^8.75^ PFU/ml, respectively. Serotype A strain was amplified in steers and this bovine-amplified stock was named A/SKR/2010-PI-BovP1, yielding a title of 10^8.40^ PFU/ml in LFBK-avb6 cells (S1 Table).

### FMDV-O contact transmission study (steers as donors)

**Fig 3:**
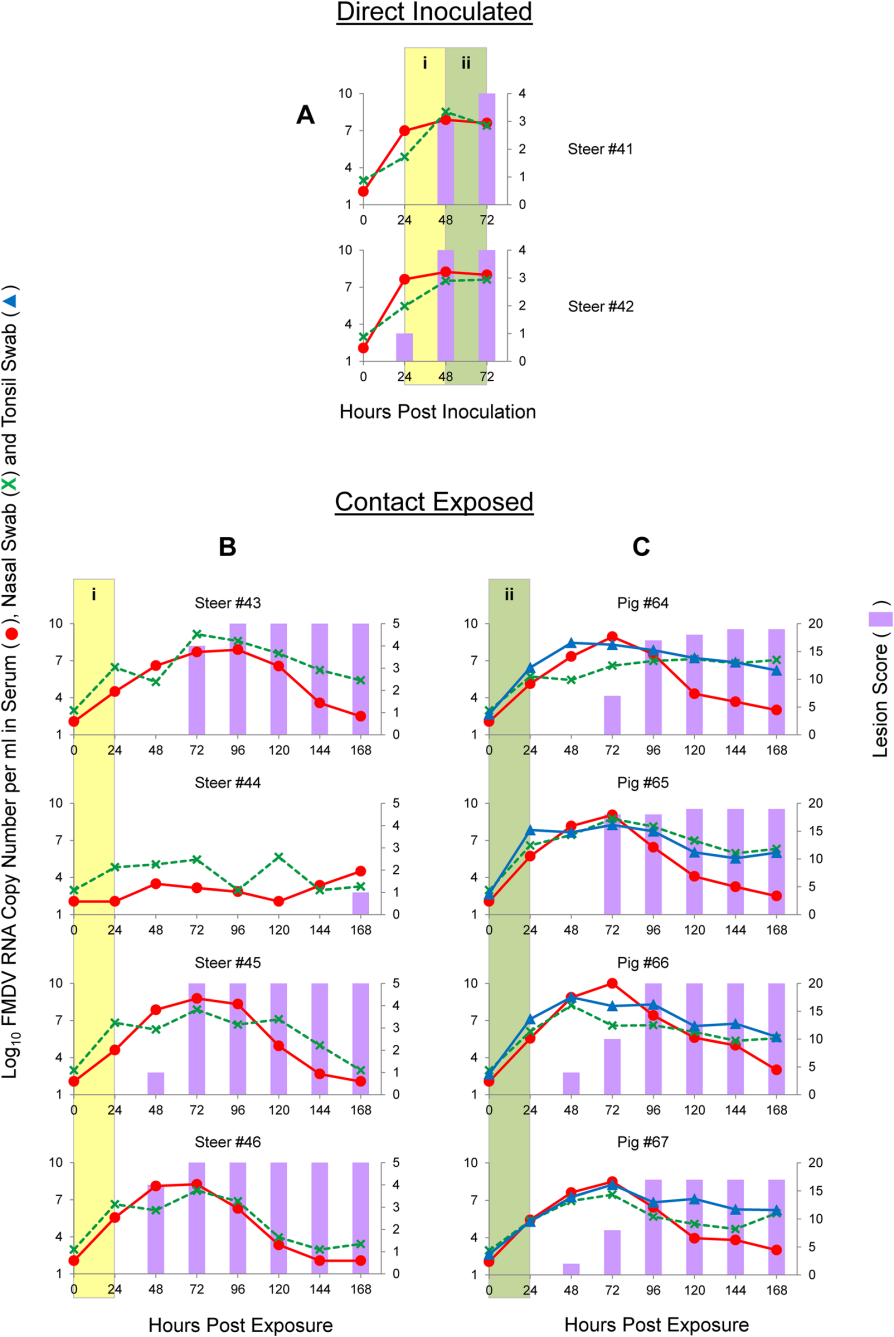
Graphs illustrating the dynamics of FMDV infection with the O/SKR/2010 strain by direct inoculation of steers (A) and contact exposure of steers (B) or pigs (C). Time on the X-axes is in hours post-inoculation (A) or hours post-exposure (B and C). Lines represent log_10_ RNA copy numbers per ml of virus expressed in the left Y-axes. Bars represent a cumulative index of FMD lesion distribution expressed in the right Y-axes. (i) Shaded yellow areas in figures A and B indicate the 24 h period that these animals were sharing the room. (ii) Shaded green areas in figures A and C indicate the 24 h period that these animals were sharing the room.

In order to study FMDV-O infection and transmission dynamics after direct inoculation and contact exposure, two donor steers (#41 and #42) were directly inoculated with 10^6.1^ PFU each of O/SKR/2010-PI-BovP1 by the intraepithelial lingual route. This direct inoculation led to severe, rapid and synchronous FMD in both steers (Fig 3, panel A). Both DI animals were viremic and had FMDV RNA in nasal swabs during the contact periods, from 24 to 72 hpi, with peaks of FMDV RNA at approximately 48 hpi. Both animals had inoculation site vesicles at 24 hpi and secondary replication sites (away from the head) at 24–48 hpi, with maximum scores achieved at 48–72 hpi. These two DI steers were used as donors for a 1:2 ratio, steer-to-steer, time-limited contact exposure, which started at 24 hpi and lasted for 24 h (Fig 1; Fig 3, yellow areas in panels A and B). At the time of separation from donors (48 hpi, 24 hpe) all four CE steers had FMDV RNA in nasal swabs and three of them (#43, #45 and #46) were already viremic. The 3 steers that were viremic at 24 hpe developed severe, rapid and synchronous disease, with clinical signs (i.e., vesicles) starting at 48 or 72 hpe, with maximum score reached 24 h later, viremia lasting from 24 to 120–144 hpe, peaking at 72–96 hpe, and FMDV RNA in swabs from 24 hpe to the end of the evaluation period. The fourth steer (#44) had no clinical signs of FMD until 168 hpe, with lower and delayed values of viremia and FMDV RNA in nasal swabs. Utilizing the methodology described previously by Fray et al [20] it was subsequently determined that this steer had endogenous systemic type I/III IFNs at the time of inoculation which may explain the apparently low virulence (results not shown).

Subsequent to steer-to-steer contact, the same two DI steers were used for 24 h contact exposure to pigs starting at 48 hpi and maintaining 1:2 steer-to-pig ratio. (Fig 1; Fig 3, green areas in panels A and C). At the time of separation from donors (72 hpi, 24 hpe), all four CE pigs were already viremic and had FMDV RNA in nasal and tonsil swabs. All four CE pigs also had severe, rapid and synchronous FMD, with clinical signs (i.e., vesicles) starting at 48 or 72 hpe. Maximum scores were achieved 2–3 days later with viremia detected from 24 to 144 hpe, peaking at 72 hpe. FMDV RNA was detected in swabs from 24 hpe to the end of the evaluation period, peaking at 48 or 72 hpe. Overall, contact exposure studies using steers as donors for FMDV O/SKR/2010 indicated efficient shedding and transmission to CE animals that rapidly developed severe and synchronous FMD.

### FMDV-O contact transmission study (pigs as donors)

**Fig 4:**
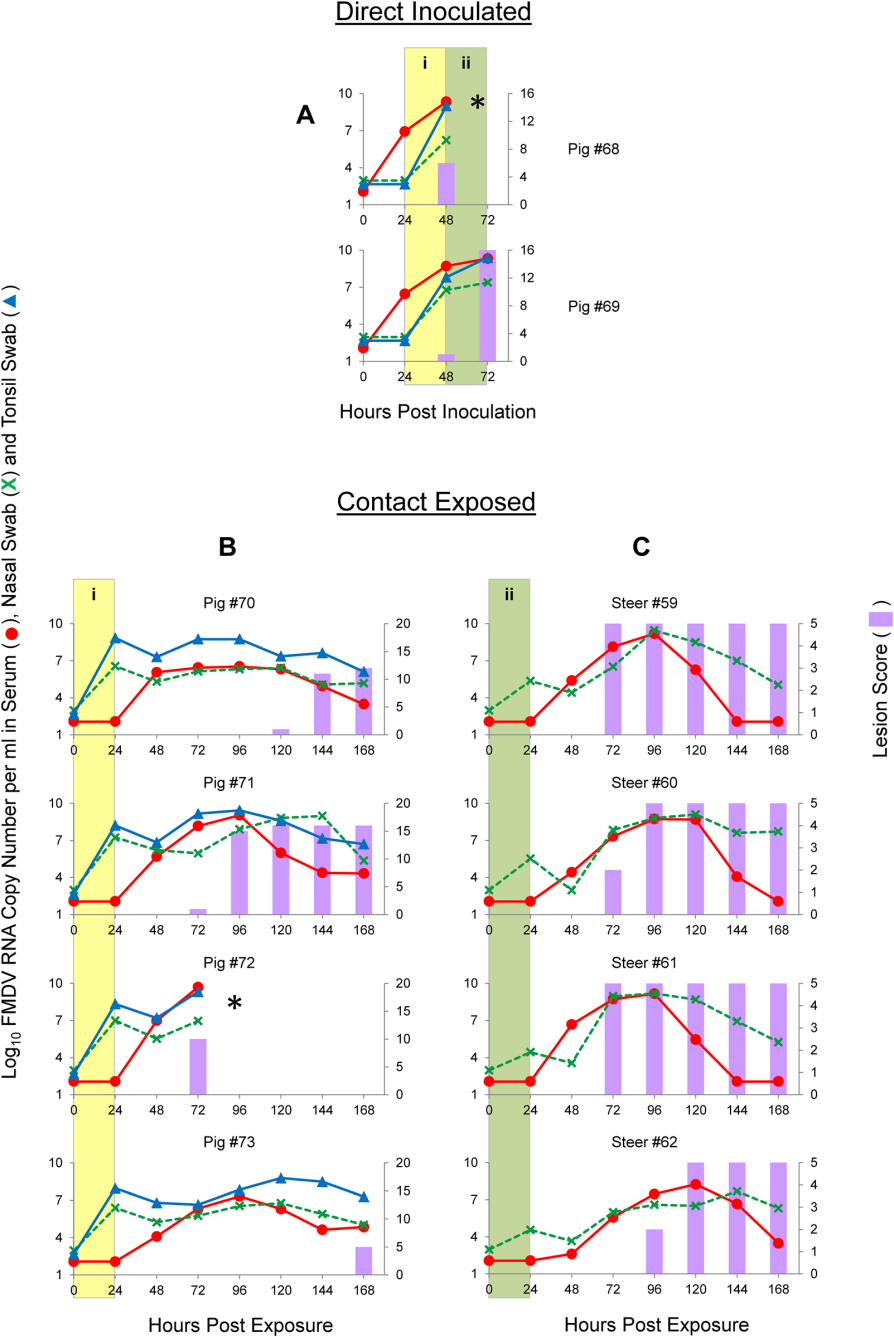
Graphs illustrating the dynamics of FMDV infection with the O/SKR/2010 strain by direct inoculation of pigs (A) and contact exposure of pigs (B) or steers (C). Time on the X-axes is in hours post-inoculation (A) or hours post-exposure (B and C). Lines represent log_10_ RNA copy numbers per ml of virus expressed in the left Y-axes. Bars represent a cumulative index of FMD lesion distribution expressed in the right Y-axes. (i) Shaded yellow areas in figures A and B indicate the 24 h period that these animals were sharing the room. (ii) Shaded green areas in figures A and C indicate the 24 h period that these animals were sharing the room. * Pigs #68 and #72 were found dead at 72 hpi and 96 hpe, respectively, due to FMD-induced myocarditis.

In order to study FMDV-O infection and transmission dynamics after direct pig inoculation and contact exposure, two donor pigs (#68 and #69) were directly inoculated with 10^5.0^ PFU each of O/SKR/2010-PI-PorP1 by the intradermal heel-bulb route. This direct inoculation produced severe, rapid and synchronous FMD (Fig 4, panel A). One of the animals died of FMDV-induced myocarditis between 48 and 72 hpi, during the pig-to-steer contact, but this did not impair the pig to steer transmission (see below). Both DI animals were viremic starting at 24 hpi and shedding FMDV RNA at the transition between the two contact periods (48 hpi). Both animals had inoculation sites vesicles at 24 hpi and secondary lesions (distant from the inoculated foot) as early as 48 hpi, with maximum scores obtained at 72 hpi for the one that survived. These two DI pigs were used as donors for a 1:2 ratio pig-to-pig time-limited contact exposure, which started at 24 hpi and lasted for 24 h (Fig 2; Fig 4, yellow areas in panels A and B). At the time of separation from donors (48 hpi, 24 hpe) all four CE pigs had FMDV RNA in nasal and tonsil swabs. These 4 CE pigs also had a synchronous duration of viremia (from 48 hpe until the end of evaluation period) and shedding of FMDV RNA (from 24 hpe until the end of the evaluation period). However the onset of clinical signs was not synchronous in these pigs, with two of them (#71 and #72) starting at 72 hpe, one (#70) at 120 hpe and the last one (#73) starting at 168 hpe. Pig #72 died of FMDV-induced myocarditis between 72–96 hpe. Clinical FMD was less severe and synchronous in these pigs compared to the pigs exposed to the same virus via DI steers.

Subsequent to pig-to-pig contact, the same two DI pigs were used for a 1:2 pig-to-steer ratio, 24 h contact exposure, which started at 48 hpi (Fig 2; Fig 4, green areas in panels A and C). Since one of the donor pigs died, the ratio was 1:4 at the end of the contact period. Nevertheless, all four CE steers also developed severe, rapid and synchronous FMD, with clinical signs (i.e., vesicles) starting at 72–96 hpe, reaching maximum score no later than 24 h thereafter. All 4 had low level FMDV RNA in nasal swabs at 24 hpe (end of contact period), with decreased FMDV RNA detection at 48 hpe, but increasing thereafter. All 4 contact animals were viremic at 48 hpe, with peak viremia at 96–120 lasting up to 144–168 hpe. Overall, contact exposure studies using pigs as donors for FMDV O/SKR/2010 indicated efficient shedding and transmission to CE animals. CE steers rapidly developed severe, rapid and synchronous FMD, but a less synchronized disease pattern including lower lesion scores was found for CE pigs, confounded with FMDV-induced myocarditis in two pigs. Results are summarized in Table 1

**Table 1:** Summary of findings and comparison of FMD status after direct and contact inoculation with both strains, O/SKR/2010 and A/SKR/2010, in both species, steers and pigs.

	FMDV O/SKR/2010	FMDV A/SKR/2010
Severe, rapid and synchronous FMD after DI of steers	Yes	Yes
FMD transmission to CE steers	Yes	Partial
Severe, rapid and synchronous FMD in CE steers	Yes	Variable
FMD transmission to CE pigs	Yes	Yes
Severe, rapid and synchronous FMD in CE pigs	Yes	Yes
Severe, rapid and synchronous FMD after DI of pigs	Yes	Yes
FMD transmission to CE pigs	Yes	Yes
Severe, rapid and synchronous FMD in CE pigs	Yes	Yes
FMD transmission to CE steers	Yes	Variable
Severe, rapid and synchronous FMD in CE steers	Yes	Delayed

### FMDV-A contact transmission study (steers as donors)

**Fig 5:**
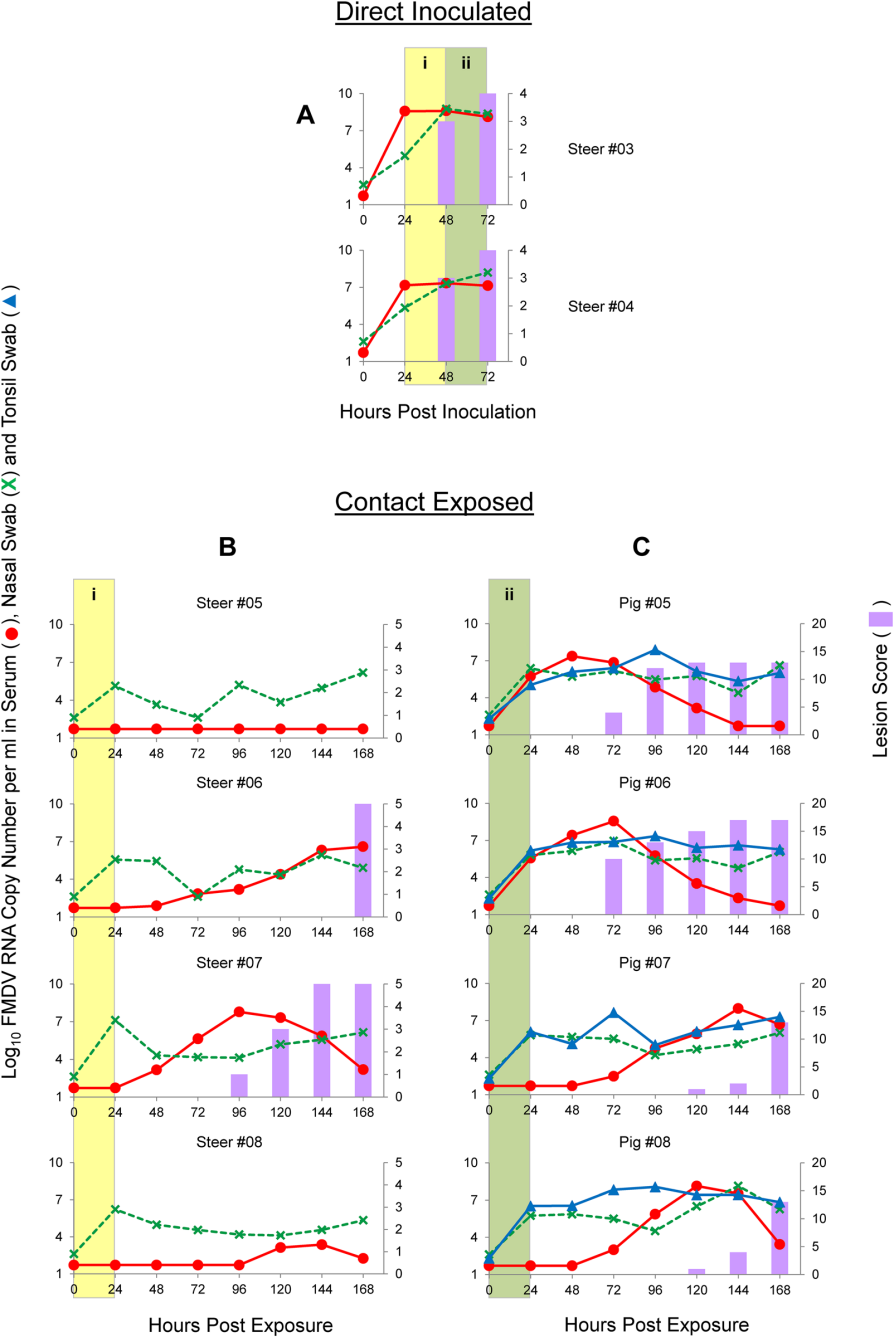
Graphs illustrating the dynamics of FMDV infection with the A/SKR/2010 strain by direct inoculation of steers (A) and contact exposure of steers (B) or pigs (C). Time on the X-axes is in hours post-inoculation (A) or hours post-exposure (B and C). Lines represent log_10_ RNA copy numbers per ml of virus expressed in the left Y-axes. Bars represent a cumulative index of FMD lesion distribution expressed in the right Y-axes. (i) Shaded yellow areas in figures A and B indicate the 24 h period that these animals were sharing the room. (ii) Shaded green areas in figures A and C indicate the 24 h period that these animals were sharing the room.

In order to study FMDV-A infection and transmission dynamics after direct inoculation and contact exposure, two donor steers (#03 and #04) were directly inoculated with 10^6.8^ PFU each of A/SKR/2010-PI-BovP1 by the intraepithelial lingual route. This direct inoculation produced severe, rapid and synchronous disease (Fig 5, panel A). Both DI animals were viremic and had FMDV RNA in nasal swabs during the contact periods, from 24 to 72 hpi, with peaks of FMDV RNA detection at 48 hpi. Both animals had inoculation site vesicles at 24 hpi and secondary replication sites (distant from the head) at 48 hpi, with maximum scores achieved at 72 hpi. These two DI steers were used as donors for a 1:2 ratio, steer-to-steer, time-limited contact exposure, which started at 24 hpi and lasted for 24 h (Fig 1; Fig 5, yellow areas in panels A and B). At the time of separation from donors (48 hpi, 24 hpe), all four CE steers had FMDV RNA in nasal swabs, which, in all steers, initially decreased before rising again before the end of the evaluation period (Fig 5B). One steer (#07) had higher values of FMDV RNA in swabs than the others at 24 hpe and was viremic at 48 hpe, with FMDV RNA in swabs detected all throughout the evaluation period and viremia lasting until the end of evaluation period. Clinical signs for this animal were detected only at 96 hpe and reached maximum score in 48 h (144 hpe). A second steer (#06) had clinical signs first detected at 168 hpe. This animal had intermittent shedding of FMDV RNA in nasal swabs and slower increasing titer of viremia starting at 72 hpe peaking at 168 hpe. The two remaining steers (#05 and #08) never developed clinical disease (i.e., vesicles) during the evaluation period. One of them (#05) had FMDV RNA in swabs but no detectable viremia whereas the second one (#08) had FMDV RNA in swabs and a delayed and low level viremia at 120–168 hpi. Because these 4 animals shared the room continuously, it cannot be determined if the last three steers got FMDV from the DI steers or from their roommates. Type I/III IFN was not detected in any of these animals at the time of inoculation (results not shown), thus indicating that preexisting antiviral activity was unlikely to be the cause of low virulence. Overall, this virus was poorly transmitted from steer to steer in the current study.

Subsequent to steer-to-steer contact, the same two DI steers were used for a 1:2 ratio steer-to-pig, 24 h contact exposure, which started at 48 hpi (Fig 1; Fig 5, green areas in panels A and C). At the time of separation from donors (72hpi, 24hpe), all four CE pigs already had FMDV RNA in nasal and tonsil swabs, and maintained high FMDV RNA values throughout the experiment. However, unlike with the serotype O virus, only two pigs (#05 and #06) were viremic at 24 hpe with vesicles appearing at 72 hpe, while the other two (#07 and #08) had a 48 h delayed viremia and clinical signs (Fig 5C). Based on clinical scores, FMD in these four pigs was severe, but poorly synchronous. Because these animals were all sharing the room it cannot be determined if FMDV transmitted to pigs #07 and #08 came from the donors or from their cohabitants, #05 and 06. Overall, contact exposure studies using steers as donors for FMDV A/SKR/2010 indicated that spread to contact animals was not as effective as it was found for O/SKR/2010, not as synchronous, and not consistently severe, but once animals got sick they ultimately developed fulminant FMD.

### FMDV-A contact transmission study (pigs as donors)

**Fig 6:**
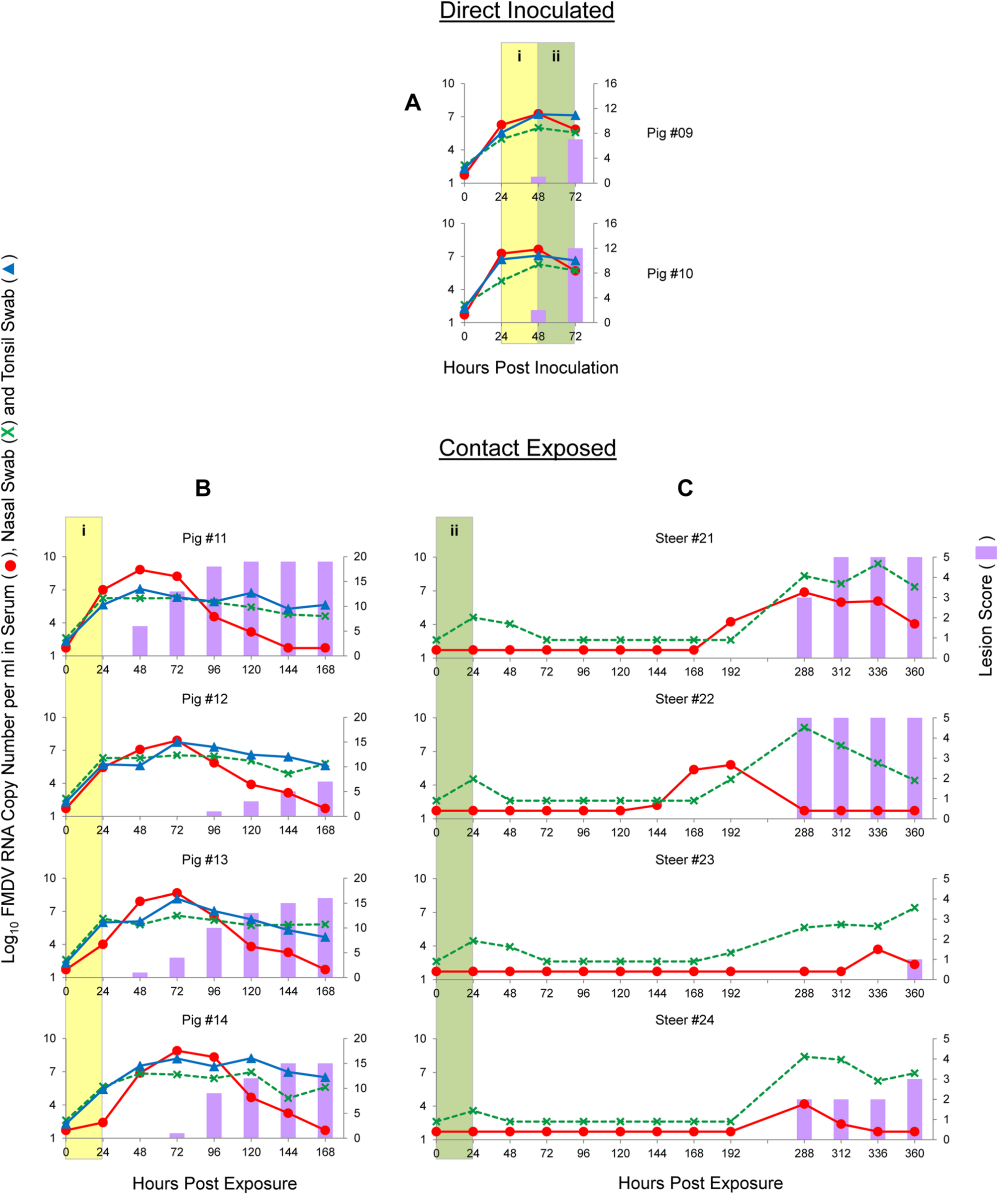
Graphs illustrating the dynamics of FMDV infection with the A/SKR/2010 strain by direct inoculation of pigs (A) and contact exposure of pigs (B) or steers (C). Time on the X-axes is in hours post-inoculation (A) or hours post-exposure (B and C). Lines represent log_10_ RNA copy numbers per ml of virus expressed in the left Y-axes. Bars represent a cumulative index of FMD lesion distribution expressed in the right Y-axes. (i) Shaded yellow areas in figures A and B indicate the 24 h period that these animals were sharing the room. (ii) Shaded green areas in figures A and C indicate the 24 h period that these animals were sharing the room. Clinical evaluation was not performed and samples were not collected from the steers between 192 and 288 hpe.

In order to study FMDV-A infection and transmission dynamics after direct pig inoculation and contact exposure, two donor pigs (#09 and #10) were directly inoculated with 10^7.8^ PFU each of A/SKR/2010-PI-BovP1 by the intradermal heel-bulb route. This direct inoculation produced severe and synchronous FMD, (Fig 6, panel A). Both DI pigs were viremic and had FMDV RNA in tonsil and nasal swabs starting at 24 hpi. Both animals had vesicles at 24 hpi at inoculation sites and at secondary replication sites (distant from the inoculated foot) as early as 48 hpi, with high scores observed at 72 hpi. These two DI pigs were used as donors for a 1:2 ratio, pig-to-pig time-limited contact exposure, which started at 24 hpi and lasted for 24 h (Fig 2; Fig 6, yellow areas in panels A and B). At the time of separation from donors (48 hpi, 24 hpe) all four CE pigs were viremic and had FMDV RNA in nasal and tonsil swabs. All four CE pigs had fulminant and synchronous FMD, with three of them (#11, #13 and #14) starting with clinical score at 48–72 hpe, and one (#12) at 96 hpe.

Subsequent to pig-to-pig contact, the same two DI pigs were used for a 1:2 ratio pig-to-steer 24 h contact exposure, which started at 48 hpi (Fig 2; Fig 6, green areas in panels A and C). All four CE steers had FMDV RNA in their nasal swabs at the end of contact time (24 hpe), however FMDV RNA dropped below the limit of detection by 72 hpe in all animals. No FMDV RNA was detected in any animal until 144 hpe (Fig 6, panel C, steer #22). Between 168–192 hpe 3 steers became viremic or shed FMDV RNA and FMDV RNA was detected in blood and swabs in the fourth steer at 288 hpe. Three steers had clinical FMD at 288 hpe and the fourth at 360 hpe. Systemic type I IFN was undetectable in these animals from the time of inoculation until the onset of viremia (results not shown). Thus pre-exisiting antiviral effect seems not to be the cause of the observed low virulence. Overall, contact exposure studies using pigs as donors for FMDV A/SKR/2010 indicated that FMD readily spread between pigs that all had fulminant and synchronous disease. A completely different, atypical disease pattern was observed in steers, in which subclinical infection and clinical FMD were substantially delayed. Results are summarized in Table 1.

### Statistical analysis of clinical scores and FMDV RNA detection in serum and nasal swabs

Three parameters (clinical score data, FMDV RNA quantified in serum and FMDV RNA quantified in nasal swabs) were used to compare experimental groups (results shown in S1 Fig). No significant differences (p-values > 0.05) were found for DI animals when compared by time-point post inoculation (24–48 hpi vs. 48–72 hpi), species (bovine vs. porcine) or serotype (O vs. A). CE animals were compared by serotype (O vs. A), donor species (bovine vs. porcine), or recipient species (bovine vs. porcine). When comparisons were made for experiments that involved serotype O, statistically significantly differences were found only between clinical scores of CE pigs exposed to *steers* (Fig 3C) and CE pigs exposed to *pigs* (Fig 4B), as well as between CE *pigs* exposed to pigs and CE *steers* exposed to pigs (Fig 4C). No statistically significant differences were found for other parameters with serotype O alone. In experiments that involved serotype A, statistically significant differences were found for all three parameters between CE *pigs* exposed to pigs (Fig 6B) and CE *steers* exposed to pigs (Fig 6C), for FMDV RNA in serum between CE pigs exposed to *pigs* (Fig 6B) and CE pigs exposed to *steers* (Fig 5C), and for FMDV RNA in nasal swabs between CE steers exposed to *steers* (Fig 5B) and CE steers exposed to *pigs* (Fig 6C), as well as between CE *steers* exposed to steers (Fig 5B) and CE *pigs* exposed to steers (Fig 5C).

When comparisons were made between all CE animals of a species exposed to different serotypes (O vs. A), statistically significant differences (in all three parameters) were found only for CE steers, not for CE pigs. In addition, for each CE animal, considering the first day with a clinical score >1 as the onset of clinical disease, a mean time to onset (in days) and a 95% confidence interval for that mean were calculated. Table 2 shows that the time to onset was not significantly different between the serotype O-exposed groups (all confidence intervals overlapped substantially) whereas for serotype A-exposed groups the time of onset of clinical disease was significantly longer for in-contact steers than for in-contact pigs.

**Table 2:** Mean time (in days) and 95% confidence interval of onset of FMD in CE animals for serotypes O and A.

DI species	CE species	Serotype O	Serotype A
cows	cows	2.3±0.7	6.8±1.9*
cows	pigs	2.5±0.6	4.0±1.1
pigs	pigs	4.5±1.9	2.8±1.9
pigs	cows	3.3±0.5	>8*

* For these groups, the time to onset was significantly longer.

### FMDV RNA detection in room air

FMDV shedding was monitored by air sampling in selected rooms housing DI and/or CE pigs throughout the length of the experiments, in selected rooms (Fig 1A–1B and Table 3). In each experiment when bovine were the donors, a single sampling device was operated in the room in which CE pigs were housed. In each experiment when pig were donors, the sampling device was operated in the room in which DI pigs were inoculated and later on CE pigs were housed. All experiments yielded generally similar temporal patterns of detection and mean concentrations of FMDV RNA in a range of 3.40 to 4.63 log_10_ RNA CN per 1000 l (Table 3).

**Table 3:** Detection of FMDV RNA (log_10_ RNA copies/1000 l) in room air samples.

	Steer to pig (room B in Fig 1)				Pig to pig (room A in Fig 2)			
Time period, hpe ^(a)^	Number of DI steers in the room	Number of CE pigs in the room	FMDV O/SKR/2010, RNA CN in air ^(b)^	FMDV A/SKR/2010, RNA CN in air	Number of DI pigs in the room	Number of CE pigs in the room	FMDV O/SKR/2010, RNA CN in air	FMDV A/SKR/2010, RNA CN in air
-24 to 0	0	4 ^(c)^	Negative	Negative	2 (0 to 24 hpi ^(d)^)	0	Negative	3.32
0 to 24^(e)^	2 (48 to 72 hpi)	4	3.45	3.91	2 (24 to 48 hpi)	4	4.75	5.11
24 to 48	0	4	4.43	4.86	0	4	4.43	5.22
48 to 72	0	4	5.82 ^(f)^	4.26	0	4	5.00	5.91
72 to 96	0	4	5.65	4.70	0	3 or 4^(g)^	3.29	5.08
96 to 120	0	4	3.52	3.87	0	3 or 4	2.66	4.60
120 to 144	0	4	2.91	5.30	0	3 or 4	2.60	4.35
144 to 168	0	4	1.70	3.89	0	3 or 4	2.20	3.95
Mean ^(h)^			3.66	4.37			3.40	4.63

^(a)^ Hours post exposure by direct contact,

^(b)^ Corresponds to FMDV RNA, detected in the air filters, collected every 24 h, at the end of the time period.

^(c)^ During this period these animals were not exposed to virus yet.

^(d)^ Hours post inoculation of donors.

^(e)^ Period that contact exposure occurred.

^(f)^ Underlined text indicates the minimum (different than 0) and maximum values detected for each room.

^(g)^ for serotype O one of the pigs died of FMDV induced myocarditis at the end of the 48–72 hpe period, then only 3 pigs remained in the room.

^(h)^ Geometric means were calculated only from samples from which FMDV RNA was detected.

**Fig 7:**
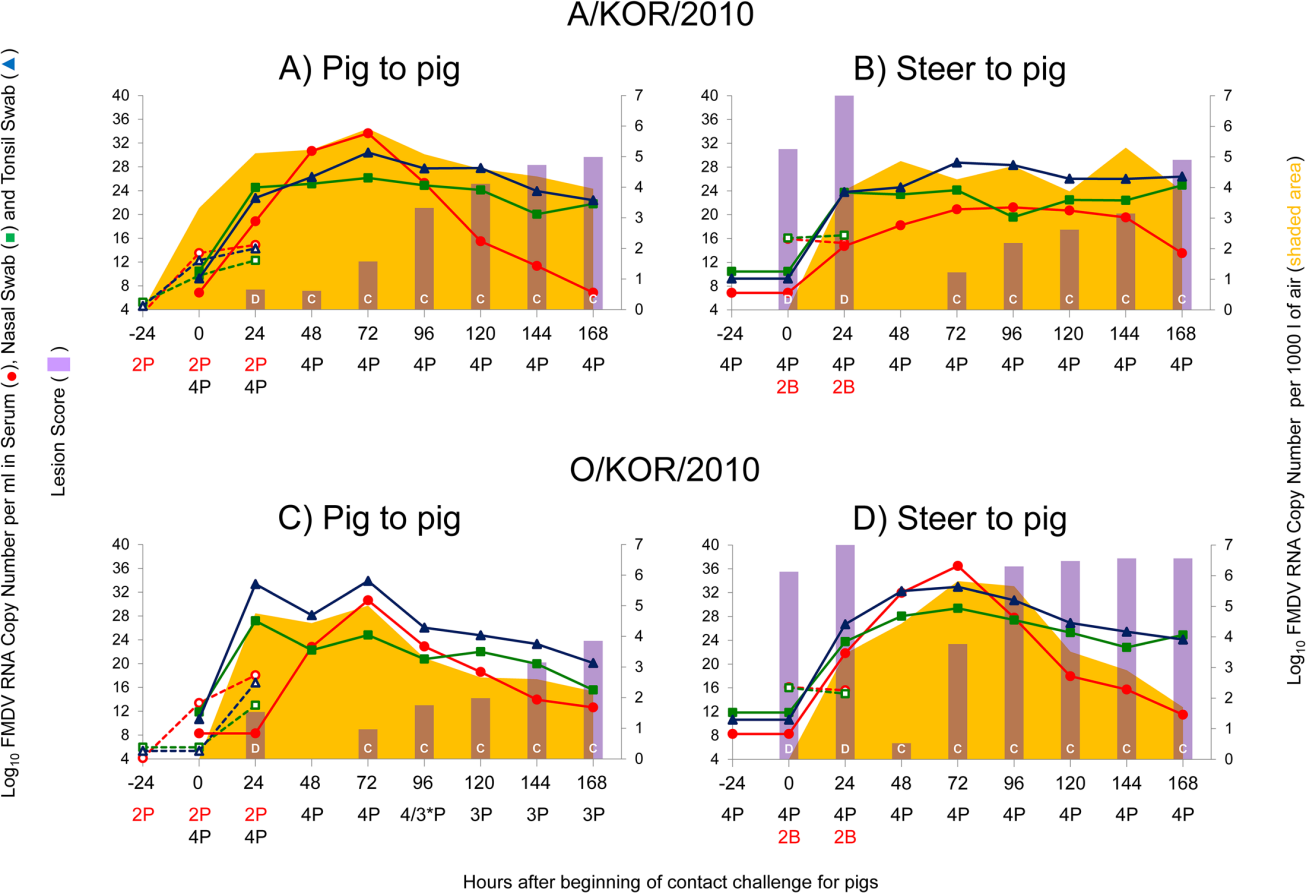
Relationship between detection of FMDV in air samples and animal samples in the context of clinical scores of animals directly- or contact-inoculated with strains A/SKR/2010 (top panel) and O/SKR/2010 (bottom panel). X-axes describes primarily the parameters of the disease in CE pigs. In consequence, time point 0 on the X-axes corresponds to beginning of contact exposure of CE pigs. Regarding time points for donor animals, for Fig 7A and 7C, 0 hpc corresponds to 24 hpi of donor pigs and for Fig 7B and 7D, 0 hpc corresponds to 48 hpi of donor steers. Lines represent cumulative levels (sum of all animals) of FMDV RNA, expressed as log10 genome copy numbers per ml in the left Y-axes. Dotted lines correspond to DI animals whereas continuous lines correspond to CE animals. Bars represent the average of lesion score of animals, scaled to fit on the 4–40 Y-left axis. Bars with a “D” correspond to directly inoculated animals whereas bars with a “C” correspond to contact exposed animals. FMDV detected in air (shaded area), expressed as log10 genome copy numbers per 1000 liters, is shown on the right Y-axes. Numbers/letters below time points correspond to the number and species (P for porcine, B for bovine) of animals present in the room during air sampling. Red font is for DI animals whereas black font is for CE animals. * indicates one of the CE pigs died of FMDV induced myocarditis between 72 and 96 hpe.

The earliest time period that FMDV RNA was detected in air (3.32 log_10_ FMDV RNA CN per 1000 l) was 0 to 24 hpi in the room housing two DI pigs inoculated with A/SKR/2010 (time point 0 hpe in Fig 7A). This coincided with first detection of FMDV RNA in swabs and serum of both DI animals (24 hpi in Fig 6A, empty markers at 0 hpe in Fig 7A) and preceded the detection of clinical signs by 24 h. At the moment of collection of this filter, the room contained only two DI pigs (24 hpi in Fig 2). For the following data point, collected after 24 h of two DI and four CE pigs sharing the room, there was approximately 2 log_10_ greater detection of FMDV RNA (time point 24 hpe in Fig 7A, Table 3). At the moment this filter was collected (48 hpi in Fig 6A and 24 hpe in Fig 6B, empty and full markers at 24 hpe in Fig 7A) all pigs were viremic and shedding virus, but only DI pigs had clinical signs (bars at 48 hpi in Fig 6Aand at 24 hpe in Fig 7A). After removal of DI pigs and until the end of the evaluation period (168 hpe), the amount of FMDV RNA detected in the air was within +/- 1 log_10_, peaking at 72 hpe, with gradually decreasing detection thereafter (Fig 7A, Table 3). Detection of FMDV RNA in swabs followed a similar pattern, but distinct from viremia or clinical scoring (Fig 7A).

For the steer-to-pig A/SKR/2010 transmission experiment, no infected animals were inside the room until 0 hpe (48 hpi in Fig 1), therefore the first FMDV RNA detection in air (3.91 log_10_ FMDV RNA CN per 1000 l) was at 24 hpe (Fig 7B), coinciding with FMDV RNA detection in swabs of all six animals, viremia in four of the six pigs (except pigs # 07 and 08), and vesicles in DI animals only (72 hpi in Fig 5A and 24 hpe in Figs 5C and 6B). All consecutive data points were within +/- 1 log_10_ until the end of the evaluation period (168 hpe), similar to what occurred for the previous A/SKR/2010 experiment described above, but now with three peaks of virus in air instead of one, at 48, 96, and 144 hpe. This difference may be related to the less synchronous disease experienced by these CE pigs, as shown in Fig 5C. It is noteworthy that substantial RNA was detected in air at 48 hpe, a collection point at which the only animals in the room for the last 24 h were pre-clinical pigs.

For O/SKR/2010 pig-to-pig, at the moment of the first air sampling interval, the DI pigs were still not shedding virus in swabs and had neither vesicles nor FMDV RNA detection in the sampled air, despite the fact they were viremic during this period (24 hpi in Fig 4A and 0 hpe in Fig 7C). FMDV RNA was first detected in air (4.75 log_10_ FMDV RNA CN per 1000 l) 24 h later (at 48 hpi), at the end of the contact period, simultaneously with FMDV RNA in swabs in all six pigs, but viremia and vesicles detected in DI pigs only (Fig 4A, 24 hpe in Figs 3B and 6C). Values stayed in within +/- 1 log_10_ until 72 hpe, with a slight decrease thereafter (Fig 6C), which coincided with the loss of one pig at this time point (Fig 4C). It is noteworthy that substantial RNA was detected in air at 48 hpe, a collection point at which the only animals in the room during the prior 24 h were pre-clinical pigs. Similar results were obtained for the second O/SKR/2010 experiment (steer-to-pig), where first detection of virus in air (3.45 log_10_ FMDV RNA CN per 1000 l) occurred at the end of the exposure period (24 hpe in Fig 7D). At this time point, DI steers and CE pigs were shedding virus in swabs and were viremic, but only DI steers were positive for clinical score (72 hpi in Fig 3A and 24 hpe in Figs 3C and 6D). Peak of FMDV RNA detection in serum, swabs and air filters occurred simultaneously 48 h later (72 hpe in Fig 7D), decreasing thereafter at an approximate rate of 1 log_10_ a day until the end of the evaluation period (Fig 7D). FMDV RNA in swabs was highly positively correlated with viral RNA in air samples, for both strains and swab types, with the exception of the serotype A steer-to-pig group (Fig 7D, Table 4)

**Table 4:** Association between virus shedding and its detection in room air.

FMDV strain	Study	Nasal swabs	Tonsil swabs
A/SKR/2010	Pig to pig	r +0.85	r +0.71
A/SKR/2010	Steer to pig	r ≤0.10	r ≤0.10
O/SKR/2010	Pig to pig	r +0.84	r +0.95
O/SKR/2010	Steer to pig	r +0.79	r +0.91

## Discussion

It has been extensively described that most FMDV strains have a broad host range. However, there are a few reports in the literature describing species-specificity of FMDV under field and/or laboratory conditions. One of the most cited examples is the 1997 FMD outbreak in Taiwan. This strain, O/TAW/97, devastated the country’s pig industry but did not spread to cows; the restricted host range was confirmed when assayed in cattle under laboratory conditions [3, 21]. Two other examples of limited host range under field conditions occurred in South Korea in the early 2000s. The outbreak in the year 2000 (caused by FMDV O/SKR/2000, PanAsia strain, ME-SA topotype), affected Korean native and dairy cattle but not pigs in the field. Interestingly, this strain caused clinical FMD in experimentally inoculated Holstein cattle and pigs [22–24]. The following outbreak in South Korea in the year 2002 (strain O/SKR/2002, also belonging to the PanAsia strain) affected pig farms and one single cow. Similar results were obtained in experimental inoculations; pigs developed FMD but cows did not [7, 25]. A clear explanation was never found for these differences in host range of these two closely related isolates [25].

During 2009–2010, two endemic lineages (serotype A and O) of FMDV spread from Southeast Asia to eastern Asia causing outbreaks in 6 countries in the region, including South Korea. The serotype A affected mainly cows while the serotype O affected pigs, wild boar, cattle, buffalo, small ruminants and gazelles [26, 27]. In the investigations presented herein, in order to determine that clinical results correlate with the differential host susceptibility described in the field, we compared two 2010 isolates from South Korea, one from each serotype, for their virulence and infection dynamics in bovine and porcine either after direct inoculation or infection by exposure by intra- or inter-species direct contact.

Our findings demonstrated that O/SKR/2010 readily infected pigs and steers and caused a homogeneous pattern of typical signs of FMD in both species. This strain was also easily transmitted by direct contact within and between species. This is consistent with field observations during the outbreaks where both pigs and cattle were affected [12, 28]. All these O/SKR/2010 experimentally-infected animals had a severe, rapid and synchronous disease, characterized by similar FMDV RNA detection in nasal swabs and serum at 24–48 hpe and clinical signs in most animals at 24–96 hpe with none later than 168 hpe. The time course and infection dynamics are consistent with the animals being infected during the 24 h exposure to DI donors. These results indicate that laboratory-generated data with O/SKR/2010 was well-aligned with the field reports, wherein pigs or cattle got the disease, and it was easily and promptly transmitted throughout most of the country.

Contrastingly, data from experimental inoculations with A/SKR/2010 were not consistent with findings from the field. During the field outbreak, cows were affected and pigs were not [11]; however, under experimental conditions most steers exposed to this strain by direct contact had delayed or no disease. Furthermore, it could also be argued that some of the CE steers became infected by roommates and not necessarily from DI donors, thus further diminishing the extent of transmission from the intended donors. Such phenomena have been previously described [3, 29]. Overall, the transmission patterns of the serotype-A virus to cattle were markedly different from that seen with FMDV serotype-O in which there was clear evidence of efficient to-steer transmission.

Surprisingly, and in contrast to field cases, pigs had rapid, severe and contagious disease when infected with A/SKR/2010 by direct inoculation or contact exposure, despite the fact that the inoculum was a bovine-derived virus, which apparently did not diminish the effect of the FMD clinical or virological progression in pigs. Overall, the clinical syndrome was very similar to the clinical syndrome of the serotype O virus. These results clearly show that under experimental conditions host-range for this A/SKR/2010 strain is distinct from what was seen in the field. Furthermore, specific virus-host interactions that made this strain inefficient to transmit from cattle or pig to cattle may explain why the serotype A virus was not thoroughly distributed during the outbreak. The lower virulence in cattle may have resulted in less virus spread resulting in a decreased probability of pigs being exposed during the short lived outbreak. In addition, the lack of infection of pigs in the field may also be explained by prompt and complete eradication of animals in affected farms on the day of FMD confirmation together with different husbandry practices between cattle and pig farms, as was described for the outbreak that occurred in 2000 [22]. Furthermore, the genetic background of animals available in South Korea may be different from the breeds of pigs used in the experiments described herein. However, the differences between serotypes found between pigs of same genetic background are not compromised by this.

The concept of this Korean serotype A strain being less virulent in pigs was disproved in the current experiments. Various types of studies can be done to characterize the ability of different FMDV strains to transmit and infect animals under controlled direct contact exposure. It is our experience that such findings from controlled experiments may be utilized to interpret data from field outbreaks wherein some strains are described as more infectious than others or with limited host range. The results we present here demonstrated that controlled challenge experiments are useful to determine the biological properties of emerging strains of FMDV.

For designing these studies and analyzing the results it is assumed that infectiousness of donors was constant throughout the contact exposure periods and the infection/disease process was similar for both serotypes. In order to minimize use of live animals, we designed studies that utilized the same donors in sequential exposure periods to both species. The sequential exposure periods always occurred with intra- species first followed by inter-species in order to minimize potential effect of viruses evolving in more than one host species. Relatively high doses of FMDV were used to infect the donor animals in order to assure rapid synchronous disease resulting in more efficient exposure [29, 30]. The dose of FMDV received by each contact animal was not measurable, but judging by the clinical outcome of DI animals during the period of contact we can extrapolate that all contact animals were exposed to enough FMDV during the 24 h contact to cause full blown infection and clinical disease.

Pigs have been described as having a critical role as donors in the airborne transmission among different premises during FMDV outbreaks [31]. Additionally, the strain of virus markedly influences the amount of airborne virus emitted [1, 15, 32, 33]. In the current study, we conducted air sampling in rooms that held pigs to determine if the amount of virus shed in air was associated with different disease patterns. We showed that there is an association between virus detected in air and virus shed by pigs (detected in nasal and tonsil swabs) that is strain independent. Similar detection of FMDV RNA in air samples from experiments with either serotype suggests that aerobiological properties of these FMDV strains was not critical in determining the observed differences in the properties of these viruses in the field. This contrasts previous findings from our laboratory [15], in which we found differences of 10–100 fold in detection of viruses of serotypes O and A in air samples. The comparison of the current and previous results suggests the need to study each particular strain individually to understand its intrinsic characteristics rather than making serotype-wide generalizations based on limited experiments or field history of the outbreaks.

## Conclusions

In the current study, under controlled conditions, O/SKR/2010 was readily transmissible to both bovine and porcine sentinels, regardless of the donor source species of virus. In contrast, the A/SKR/2010 strain was readily transmissible to pigs, but transmission to steers was less efficient. This lack of cattle transmission together with prompt and effective control measures may explain the reason why the A/SKR/2010 outbreak had limited distribution. Thus, the prediction of the probability of transmission of a particular strain of FMDV is more complex than simply using conventional proxies of transmission such as quantitative measures of shedding, viremia, and clinical scores. Temporal and quantitative trends in the detection of FMD in the environment, swabs and serum certainly contribute to the transfer; but transmission also may be determined by intractable qualities such as virulence, tropism, and virus-host interactions that required experimental inoculation to be determined. Ultimately, the only definitive test of transmissibility of a virus is through well-designed controlled transmission experiments using relevant natural host species, including those not involved in the outbreak. Additionally, it is apparent that conclusions regarding biological host range should not be made solely from epidemiological data collected from the field.

## Supporting Information

S1 Fig p-values for pairwise comparisons of FMDV RNA concentrations in serum (AUC), swabs and clinical scores by non-parametric Mann-Whitney U tests. (PDF)

S1 Table Origin and passage history of foot-and-mouth disease virus (FMDV) strains used in this study. (PDF)

S1 Text Confirmation of FMDV-induced myocarditis. (PDF)
