# Supplementary material for: Evaluation of Infectivity, Virulence and Transmission of FDMV Field Strains of Serotypes O and A Isolated In 2010 from Outbreaks in the Republic of Korea
Source: PLoS One. 2016 Jan 6;11(1):e0146445. doi: 10.1371/journal.pone.0146445 (PMC4703371; doi:10.1371/journal.pone.0146445)
Supplement: S1 Fig — (PDF) [file pone.0146445.s001.pdf]

S1 Fig. p-values for pairwise comparisons of FMDV RNA concentrations in serum (AUC) by non-parametric Mann-Whitney U tests

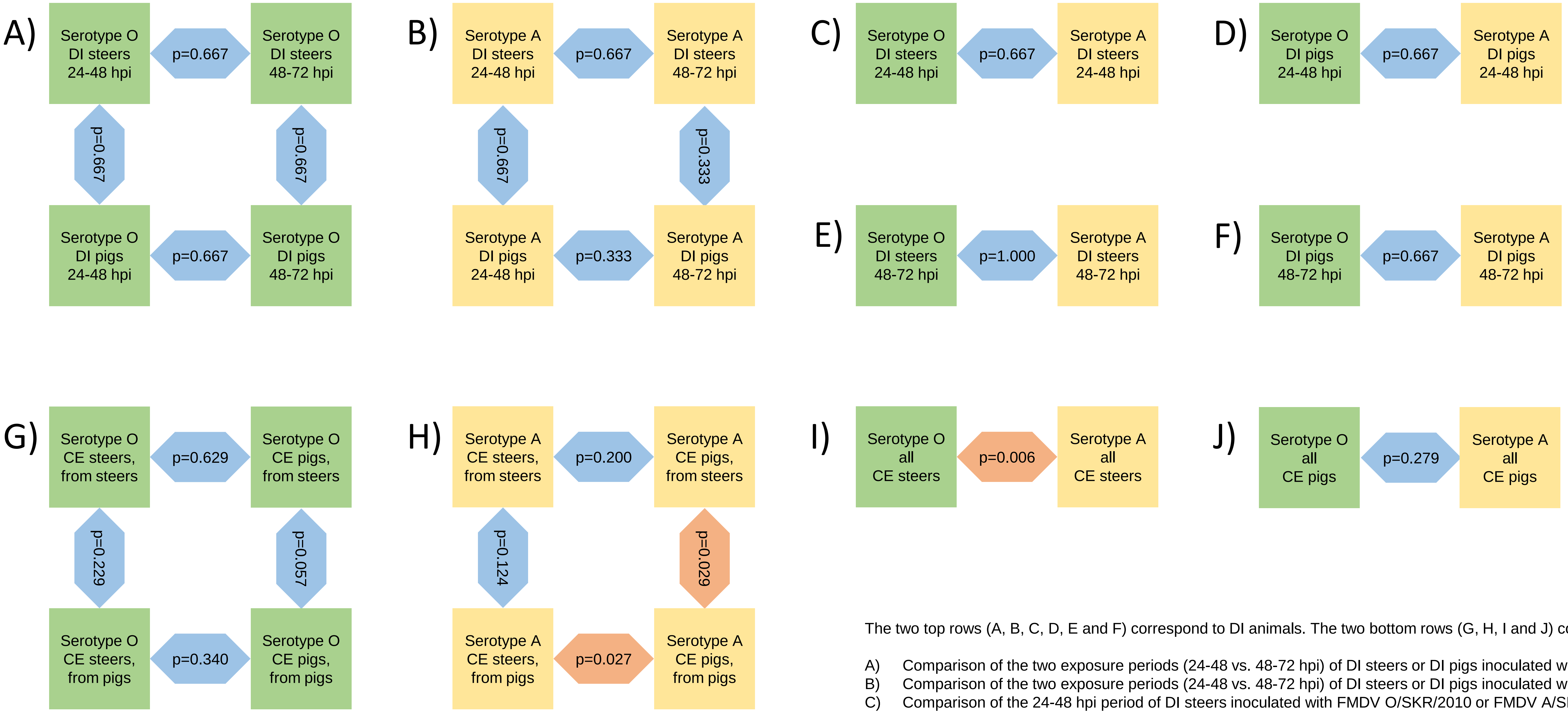

The two top rows (A, B, C, D, E and F) correspond to DI animals. The two bottom rows (G, H, I and J) correspond to CE animals.

A) Comparison of the two exposure periods (24-48 vs. 48-72 hpi) of DI steers or DI pigs inoculated with FMDV O/SKR/2010
B) Comparison of the two exposure periods (24-48 vs. 48-72 hpi) of DI steers or DI pigs inoculated with FMDV A/SKR/2010
C) Comparison of the 24-48 hpi period of DI steers inoculated with FMDV O/SKR/2010 or FMDV A/SKR/2010.
D) Comparison of the 24-48 hpi period of DI pigs inoculated with FMDV O/SKR/2010 or FMDV A/SKR/2010.
E) Comparison of the 48-72 hpi period of DI steers inoculated with FMDV O/SKR/2010 or FMDV A/SKR/2010.
F) Comparison of the 48-72 hpi period of DI pigs inoculated with FMDV O/SKR/2010 or FMDV A/SKR/2010.
G) Comparison of FMDV O/SKR/2010 exposed animals, CE steer vs. CE pigs, in contact with DI steers or pigs
H) Comparison of FMDV A/SKR/2010 exposed animals, CE steer vs. CE pigs, in contact with DI steers or pigs
I) Comparison of FMDV O/SKR/2010 vs. FMDV A/SKR/2010 CE, steers exposed by contact with DI steers and DI pigs
J) Comparison of FMDV O/SKR/2010 vs. FMDV A/SKR/2010 CE, pigs exposed by contact with DI steers and DI pigs

S1 Fig. p-values for pairwise comparisons of FMDV RNA concentrations in nasal swabs (AUC) by non-parametric Mann-Whitney U tests.

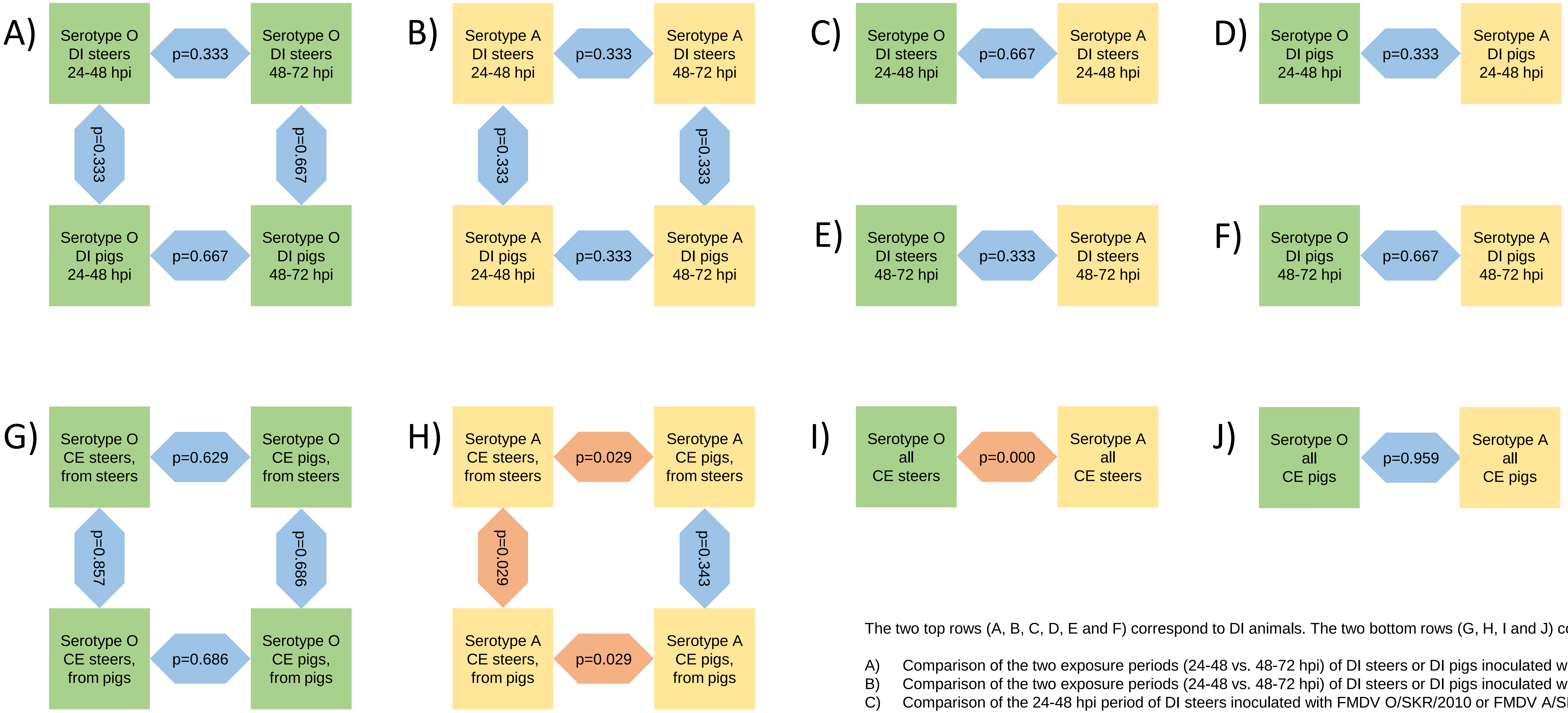

The two top rows (A, B, C, D, E and F) correspond to DI animals. The two bottom rows (G, H, I and J) correspond to CE animals.

A) Comparison of the two exposure periods (24-48 vs. 48-72 hpi) of DI steers or DI pigs inoculated with FMDV O/SKR/2010
B) Comparison of the two exposure periods (24-48 vs. 48-72 hpi) of DI steers or DI pigs inoculated with FMDV A/SKR/2010
C) Comparison of the 24-48 hpi period of DI steers inoculated with FMDV O/SKR/2010 or FMDV A/SKR/2010.
D) Comparison of the 24-48 hpi period of DI pigs inoculated with FMDV O/SKR/2010 or FMDV A/SKR/2010.
E) Comparison of the 48-72 hpi period of DI steers inoculated with FMDV O/SKR/2010 or FMDV A/SKR/2010.
F) Comparison of the 48-72 hpi period of DI pigs inoculated with FMDV O/SKR/2010 or FMDV A/SKR/2010.
G) Comparison of FMDV O/SKR/2010 exposed animals, CE steer vs. CE pigs, in contact with DI steers or pigs
H) Comparison of FMDV A/SKR/2010 exposed animals, CE steer vs. CE pigs, in contact with DI steers or pigs
I) Comparison of FMDV O/SKR/2010 vs. FMDV A/SKR/2010 CE, steers exposed by contact with DI steers and DI pigs
J) Comparison of FMDV O/SKR/2010 vs. FMDV A/SKR/2010 CE, pigs exposed by contact with DI steers and DI pigs

S1 Fig. p-values for pairwise comparisons of clinical scores (AUC) by non-parametric Mann-Whitney U tests

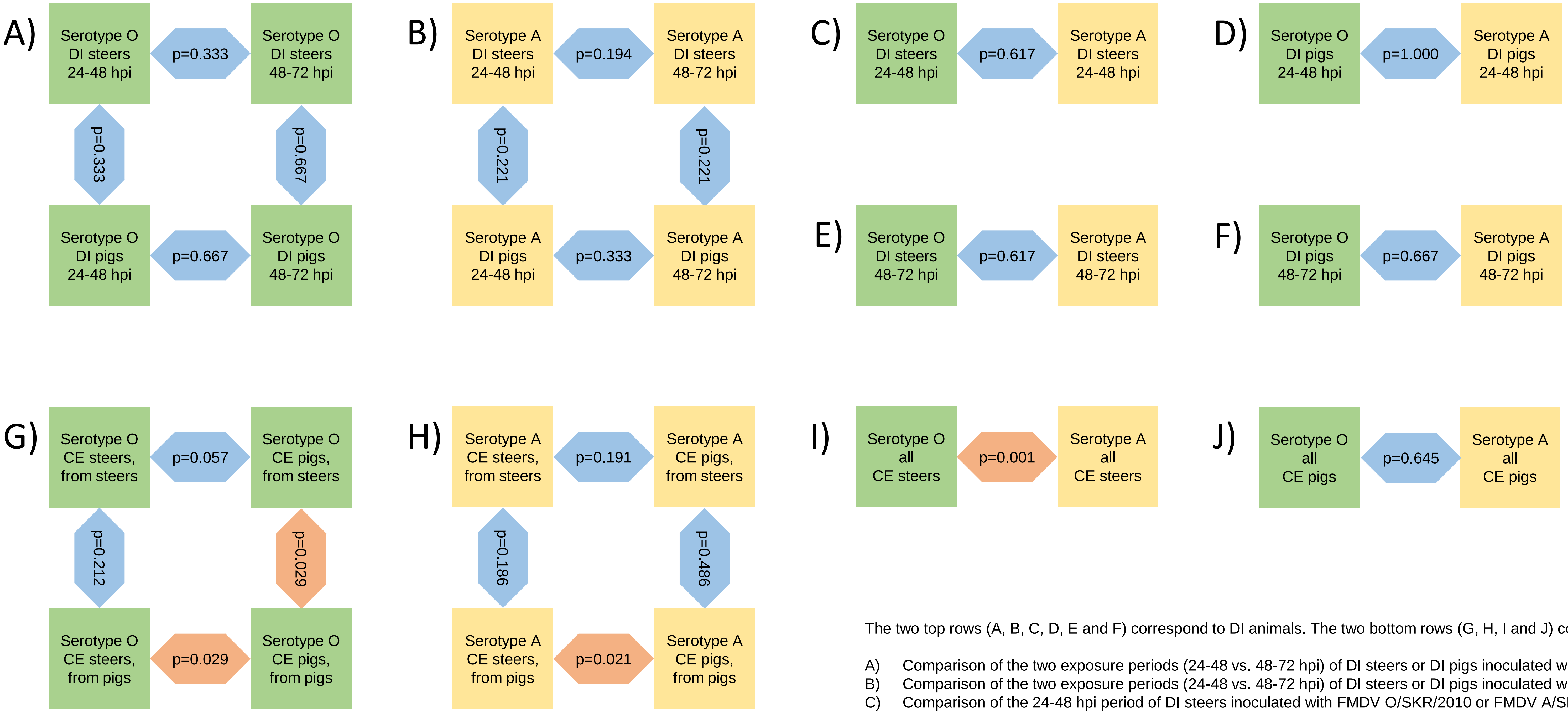

The two top rows (A, B, C, D, E and F) correspond to DI animals. The two bottom rows (G, H, I and J) correspond to CE animals.

A) Comparison of the two exposure periods (24-48 vs. 48-72 hpi) of DI steers or DI pigs inoculated with FMDV O/SKR/2010
B) Comparison of the two exposure periods (24-48 vs. 48-72 hpi) of DI steers or DI pigs inoculated with FMDV A/SKR/2010
C) Comparison of the 24-48 hpi period of DI steers inoculated with FMDV O/SKR/2010 or FMDV A/SKR/2010.
D) Comparison of the 24-48 hpi period of DI pigs inoculated with FMDV O/SKR/2010 or FMDV A/SKR/2010.
E) Comparison of the 48-72 hpi period of DI steers inoculated with FMDV O/SKR/2010 or FMDV A/SKR/2010.
F) Comparison of the 48-72 hpi period of DI pigs inoculated with FMDV O/SKR/2010 or FMDV A/SKR/2010.
G) Comparison of FMDV O/SKR/2010 exposed animals, CE steer vs. CE pigs, in contact with DI steers or pigs
H) Comparison of FMDV A/SKR/2010 exposed animals, CE steer vs. CE pigs, in contact with DI steers or pigs
I) Comparison of FMDV O/SKR/2010 vs. FMDV A/SKR/2010 CE, steers exposed by contact with DI steers and DI pigs
J) Comparison of FMDV O/SKR/2010 vs. FMDV A/SKR/2010 CE, pigs exposed by contact with DI steers and DI pigs
