# Supplementary material for: Evaluation of Infectivity, Virulence and Transmission of FDMV Field Strains of Serotypes O and A Isolated In 2010 from Outbreaks in the Republic of Korea
Source: PLoS One. 2016 Jan 6;11(1):e0146445. doi: 10.1371/journal.pone.0146445 (PMC4703371; doi:10.1371/journal.pone.0146445)
Supplement: S1 Table — (PDF) [file pone.0146445.s002.pdf]

**S1 Table: Origin and passage history of foot-and-mouth disease virus (FMDV) strains used in this study**

| Serotype | Municipality, Province | Denomination of isolate | Original host | Host inoculated [a] | Dose [b] | Denomination of high titer stock [c] | Titer [d] |
|---|---|---|---|---|---|---|---|
| O | Paju, Gyeonggi | O/PJ/KOR/2010, NVRQS10, 201012_49V | Bovine | Bovine [e] | 5.30 | O/SKR/2010-PI-BovP1 | 9.50 |
| | | | | Porcine [f] | 5.00 | O/SKR/2010-PI-PorP1 | 8.75 |
| A | Pocheon, Gyeonggi | A/Pocheon/01/KOR/2010, NVRSQ01, 201001_01V | Bovine | Bovine [e] | 5.30 | A/SKR/2010-PI-BovP1 | 8.40 |

[a] Inoculum consisted of vesicular fluid from one bovine from the outbreak site.

[b] Amount of virus inoculated, expressed as $log_{10}$ PFU, obtained in LFBK-αvβ6 cells.

[c] Nomenclature as follows: O: serotype O; A: serotype A; SKR: South Korea (Republic of Korea); 2010: year of isolation; PI: Plum Island; Bov: bovine; Por: Porcine; P1: passage 1

[d] Titer of virus obtained, expressed as $\log_{10}$ PFU/ml, performed in LFBK-αvβ6 cells.

[e] Inoculated by the intraepithelial lingual route 4 inoculation sites, 100 µl each

[f] Inoculated intradermally in the heel-bulb, 4 inoculation sites, 100 µl each
