# Supplementary material for: Evaluation of Infectivity, Virulence and Transmission of FDMV Field Strains of Serotypes O and A Isolated In 2010 from Outbreaks in the Republic of Korea
Source: PLoS One. 2016 Jan 6;11(1):e0146445. doi: 10.1371/journal.pone.0146445 (PMC4703371; doi:10.1371/journal.pone.0146445)

S1 Text. Confirmation of FMDV-induced myocarditis.

Pig R11-37 was part of the study designed to generate porcine-derived stock for FMDV O/SKR/2010. After sudden death a necropsy was performed with the following findings:

Macroscopical findings were:

- Multifocal vesicles
- Multifocal-coalescing necrotizing myocarditis.

Microscopical findings were:

- Cardiomyocyte degeneration
- Necrosis and mononuclear inflammatory infiltrate
- These findings were complemented with staining with anti-FMDV-VP1 and anti-FMDV-3D staining.

Pig R11-37, myocardium, cryosection, H&E stain

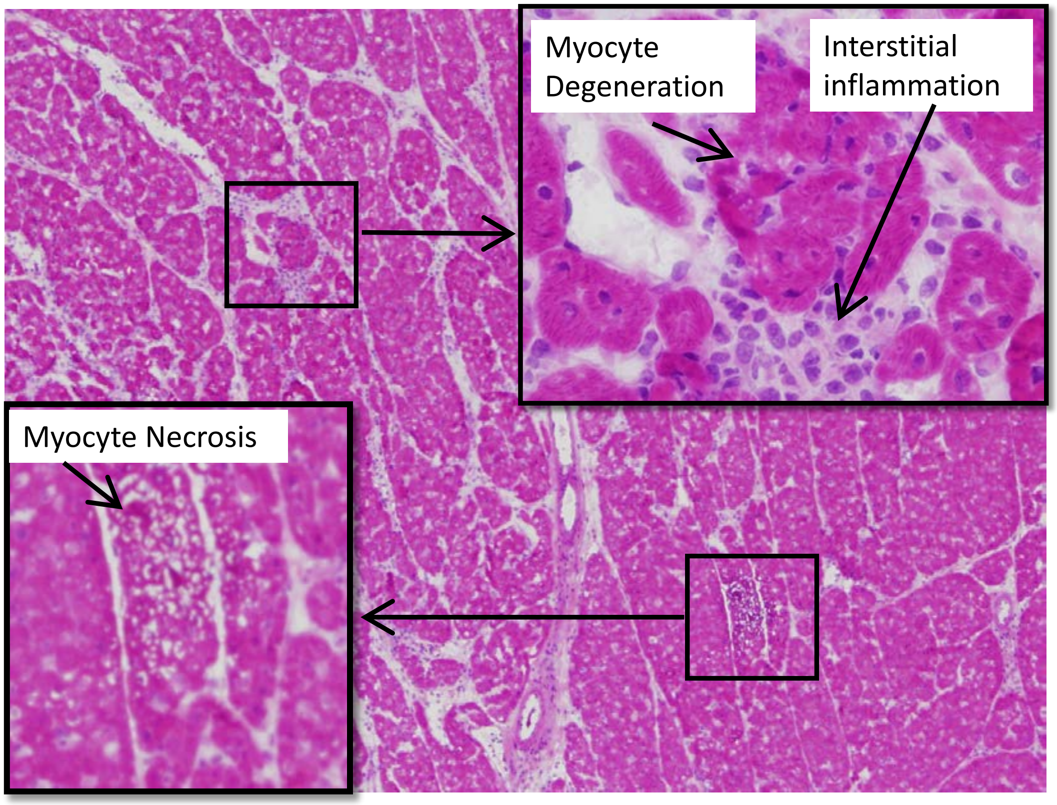

Pig R11-37, myocardium, cryosection, FMDV-specific antibody stain

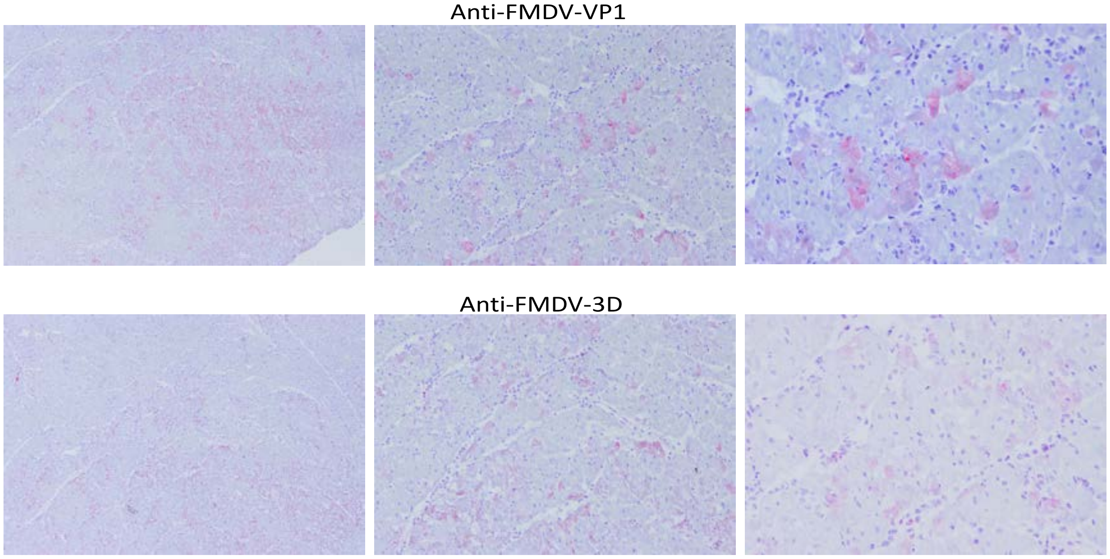

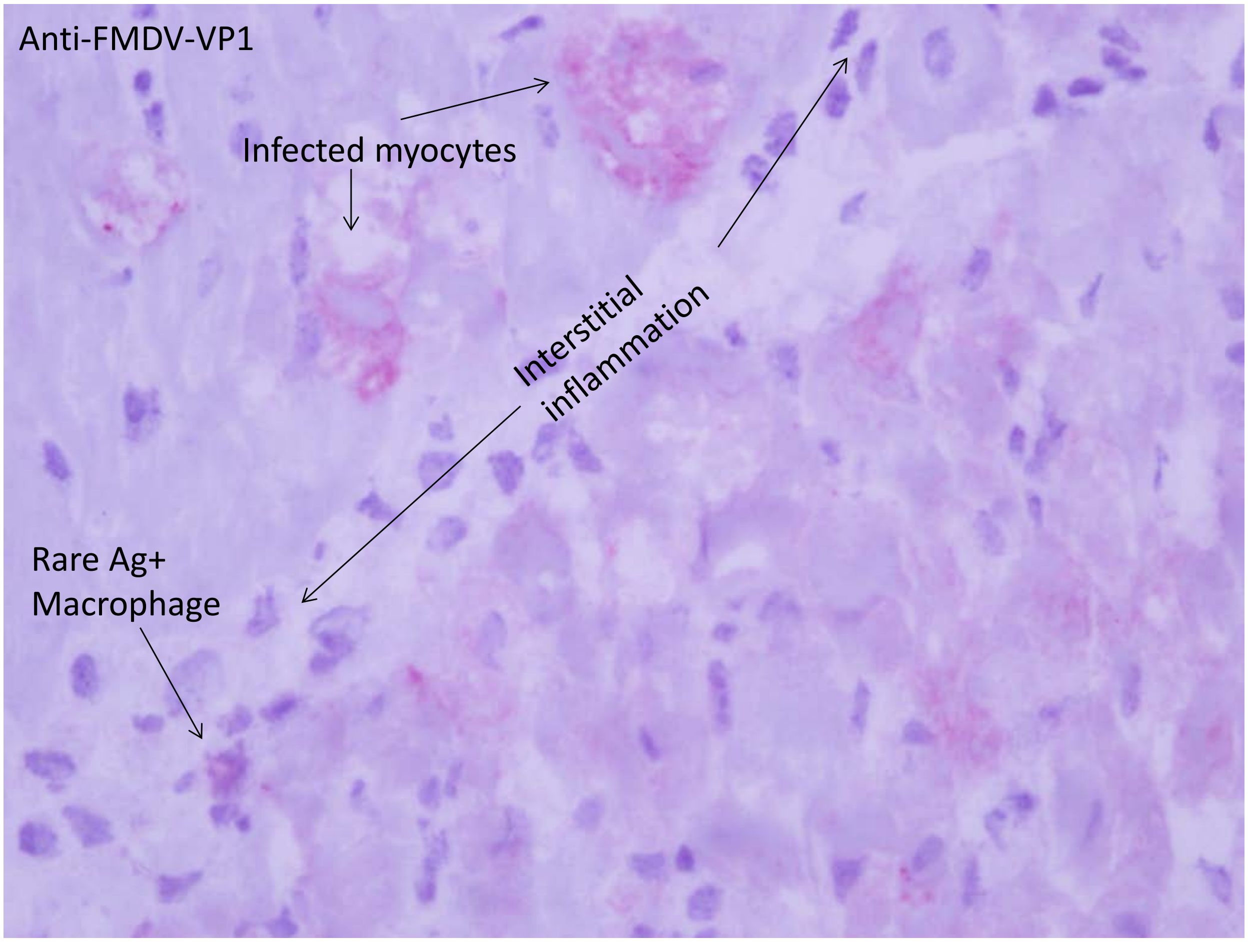

Supplement: S1 Text — (PDF) [file pone.0146445.s003.pdf]
